# Network diffusion of gender diversity on boards: A process of two-speed opposing forces

**DOI:** 10.1371/journal.pone.0277214

**Published:** 2022-11-14

**Authors:** Ricardo Gimeno, Ruth Mateos de Cabo, Pilar Grau, Patricia Gabaldon

**Affiliations:** 1 Operations Department, Bank of Spain, Madrid, Spain; 2 Business Department, Universidad CEU San Pablo, Madrid, Spain; 3 Applied Economics Department, Universidad Rey Juan Carlos, Madrid, Spain; 4 IE University, Madrid, Spain; University of Almeria: Universidad de Almeria, SPAIN

## Abstract

Network diffusion processes or how information spreads through networks have been widely examined in numerous disciplines such as epidemiology, physics, sociology, politics, or computer science. In this paper, we extend previous developments by considering a generalization of the diffusion by considering the possibility of differences in the speed of diffusion and reduction depending on the forces’ directions. In this situation, the differential speed of diffusion produces deviations from the standard solution around the average of the initial conditions in the network. In fact, this asymmetry gives rise to non-linear dynamics in which, contrary to the symmetric case, the final solution depends on the topology of the graph as well as on the distribution of the initial values. Counter-intuitively, less central nodes in the network are able to exert a higher influence on the final solution. This behavior applies also for different simulated networks such as random, small-world, and scale-free. We show an example of this kind of asymmetric diffusion process in a real case. To do so, we use a network of US Boards of Directors, where boards are the nodes and the directors working for more than one board, are the links. Changes in the proportion of women serving on each board are influenced by the gradient between adjacent boards. We also show that there is an asymmetry: the gradient is reduced at a slower (faster) rhythm if the board has less (more) women than neighboring boards. We are able to quantify the accumulated effect of this asymmetry from 2000 to 2015 in the overall proportion of women on boards, in a 4.7 percentage points (the proportion should have been an 14.61% instead of the observed 9.93% in 2015).

## Introduction

The dynamics of different types of social spreading phenomena have attracted attention among researchers of different fields. Examples of these are the studies of opinion dynamics [1], propagation of rumors [2], diffusion of fads [3], adoption of technological innovation [4, 5] and the success of some products via word-of-mouth [6–8]. A good review of different models of social spreading is [9].

When the analysis of the diffusion phenomenon is carried out using networks, a fundamental challenge is to identify the network properties and diffusion dynamics that allow information to propagate through the network. Examples of this kind of analysis that take into account network features for different processes are the studies of rumor dynamics [10], dissemination of ideas [11], the diffusion of corruption [12], gossip spreading [13] or gang crime and violence [14]. Regarding network properties, some studies characterize the topology of the networks through statistical properties of the network, such as the distribution of some centrality measures (e.g. [15–17 or 18]); other approaches make use of the adjacency matrix of the underlying network, modelling the diffusion as a Markov process ([19, 20 or 21], among others).

Moreover, most of the previously cited studies assume that there is local threshold in the fraction of neighbors that should be overtake for the spreading process to occur. However, in their analysis the connections remain fixed and only the intrinsic properties of the nodes are allowed to vary.

However, in real systems, both the properties of the nodes and connection change in time and several studies look for the conditions to achieve a consensus or an agreement within the network. Studies on network agreement use models inspired by the classical heat diffusion process. In those cases, the final outcome of the dynamic process depends on the average of the initial conditions of the variable of interest, while the network structure plays no role in how agreement emerges. This is a consequence of the way heat diffusion works: two objects with a different gradient will reduce that gradient at the same speed: the one hotter will cool at the same speed the cold one will heat. There is little room in this framework to obtain different dynamics, and if you try to add it to your network dynamics, you have to assume time-varying networks (see, [22–24]).

Asymmetric spreading of shocks through a network is a highly studied phenomenon in finance when dealing with systemics risk as financial networks often involve important asymmetries that affects the risk of contagion. [25, 26], and portfolio formation [27]. Temporal asymmetries have already been studied in [28] that show than the dynamics of processes may differ when they take place on similar networks from the static perspective. Also, the role of the peripheral nodes has been analyzed in [29] that find that the epidemic risk associated to the peripheral might be larger than their degrees values would imply, moreover, [30] conclude that in directed networks peripheral nodes without incoming links can disrupt the emergence of some collective states.

In addition, there is evidence in physical phenomena of diffusion cases, where the diffusion is asymmetric. For instance, [31] show that the diffusion through a membrane might by asymmetric by changing the shape of the pores of a membrane. This type of asymmetry has also been found for light flow depending on the properties of the semiconductor used [32]. Inspired by these phenomena, we propose a generalization of the standard diffusion models, where we allow for different speeds of diffusion depending on the sign of the variable’s gradient. We show that taking into account this slight change in the dynamics, the system brings about a nonlinear dynamical process with a final equilibrium that depends not only on the average initial values of the variable, as in the linear case, but also on the degree of the asymmetry, the topology of the graph, and the distribution of the initial conditions among nodes. To this extent, we introduce into the diffusion framework, the fact that not only network structure matters, but also the way in which agents influence each other [33]. Interestingly, this dynamic produce that the nodes that show lower centrality, as they are the last to converge to the consensus, are able to exert a higher influence on the final equilibrium than those that are more central and move faster to the generalized consensus.

To prove that this asymmetry is a real phenomenon, we show its effects in the consensus in the proportion of women that companies appoint for their boards of directors. This consensus appears as companies imitate others in their efforts to legitimize their behavior, to avoid looking as discriminating against women, but also they don’t want to be seen as disrespecting the need of hiring the best directors for the company regardless of their gender. US companies can be represented as a complex network, where the nodes are the companies, and the shared directors are the links between companies. These shared directors are conveying information on the proportion of women in other boards, and companies hence adapt their proportion of women to a similar proportion than those on their neighboring boards. However, we also show how the speed to appoint more women if you have less than your neighbors is slower that the speed to fire them when you have more than your neighbors, (i.e., there is an asymmetry in the speed of diffusion). This asymmetry makes the average proportion of women on boards being smaller than the one you would observe in the absence of the differential speed of agreement in the graph.

## A generalized diffusion process

The diffusion process we are going to consider happens in a graph *G* = (*N*, *A*) where *N* are the nodes (i.e., the individuals in the network), and *A*, is the adjacency matrix, that is, the links or edges (i.e., the interactions between individuals). Let’s consider some variable (*v*_*i*_) that represents some quantity of interest that is different for each node *i*. At each edge in the graph (*a*_*i*,*j*_ = 1) there would be a potential gradient if *v*_*i*_ ≠ *v*_*j*_. If there is a search for consensus in the network, we will expect that this gradient would decrease as times goes by (i.e., there would be an increase[decrease] in the value of *v*_*i*_ for those nodes *i* where *v*_*i*_ < *v*_*j*_[*v*_*i*_ > *v*_*j*_]). This would produce a search for a consensus that will reduce the gradient to zero (lim_*t* → ∞_|*v*_*j*_ − *v*_*i*_| = 0).

The dynamics of this search for consensus is modelled following the well-known heat diffusion process (Eq 1):

$$\begin{array}{c} \frac{dv_{i}\left(t\right)}{dt}=\sum_{j∼i}[v_{i}-v_{j}],\;\forall i=1,2,...,\;\forall j|a_{i,j}=1. \end{array}$$ (1)

In Eq 1, the value of *v*_*i*_(*t*) for each node *i* at each moment *t* will move in the direction to reduce the gradient *v*_*j*_ − *v*_*i*_. Moving from continuous to discrete time, we will have Eq 2,

$$\begin{array}{c} v_{i}\left(t+1\right)=v_{i}\left(t\right)+\eta \sum_{j∼i}a_{ij}(v_{j}\left(t\right)-v_{i}\left(t\right)), \end{array}$$ (2)

where *a*_*ij*_ is equal to one if there is an edge between nodes *i* and *j* and zero otherwise (i.e., *a*_*ij*_ is the element (*i*, *j*) in the adjacency matrix of graph *G*, 0 < *η* < 1 is the speed of correction of imbalances between period *t* and *t* + 1. Both Eqs 1 and 2, being linear, can be easily represented as matrix equations,

$$\begin{array}{c} \begin{array}{ccc} \frac{dv}{dt} & = & -L\vec{v}\left(t\right),\\ \Delta v(t) & = & -\eta Lv(t-1), \end{array} \end{array}$$ (3)

where *v*(*t*) is the vector of the values of *v* for each node *i* at every moment *t*, and *L* is the laplacian matrix (*L* = *D* − *A*; being *D* a diagonal matrix with the degree of each node, and *A* is the adjacency matrix (see [34]). An important consequence of the heat diffusion framework used in consensus analysis is that the final agreement is just the mean of the initial values for each node (lim_*t*→∞_ *v*_*i*_(*t*) = 〈*v*(0)〉).

We propose in this paper a generalization of the previous process (Eq 3), where now the speed of reduction of the gradient (i.e., *η*) is not invariant, but dependent on the sign of the gradient. To do so, we introduce the parameter *γ* < 0 as an asymmetry factor to split the two different sing of the gradient.

$$\begin{array}{c} v_{i}\left(t+1\right)=v_{i}\left(t\right)+\eta \sum_{j∼i|v_{j}\left(t\right)>v_{i}\left(t\right)}a_{ij}(v_{j}\left(t\right)-v_{i}\left(t\right))+\gamma \eta \sum_{j∼i|v_{j}\left(t\right)<v_{i}\left(t\right)}a_{ij}(v_{j}\left(t\right)-v_{i}\left(t\right)) \end{array}$$ (4)

We can consider Eq 4 as a generalization of Eq 2, since if *γ* = 1 then 4 collapse into 2. This generalized diffusion process can also be represented using a matrix framework. In order to do so, we have to consider that we are splitting an undirected network into two directional networks: *G*^*l*^ = (*N*, *A*^*l*^) and *G*^*h*^ = (*N*, *A*^*h*^). Both *G*^*h*^ and *G*^*l*^ have the same nodes *N*, but with different adjacency matrices. The first one, *A*^*l*^ represents the links from those nodes with higher values of the gradient variable than its network neighbors (i.e., $a_{ij}^{h}=1$ if *j* ∼ *i* and *v*_*j*_(*t*) > *v*_*i*_(*t*)). The second one, *A*^*h*^ represents the links from those nodes with lower values of the gradient variable than its network neighbors (i.e., $a_{ij}^{l}=1$ if *j* ∼ *i* and *v*_*j*_(*t*) < *v*_*i*_(*t*)). Note that given that in the case of a zero gradient there is no effect on *v*_*i*_(*t*) we can include those cases in either adjacency matrix without any difference in the final outcome of the consensus. Note also that the sum of both adjacency matrices is the original adjacency matrix of the undirected graph (i.e., *A* = *A*^*h*^ + *A*^*l*^).

The nodes in these directed graphs would have different degrees (i.e., *D*^*h*^ and *D*^*l*^), although once more, the addition of both degrees would be equal to the degree of the undirected graph (i.e., *D* = *D*^*h*^ + *D*^*l*^). Finally, as a consequence of having graphs with different adjacency and degree matrices, we will also have different laplacian matrices, *L*^*h*^ = *D*^*h*^ − *A*^*h*^ and *L*^*l*^ = *D*^*l*^ − *A*^*l*^. Since the sum of both directional degree matrices and adjacency matrices produce the undirected degree and adjacency matrices, if we sum both directed laplacian matrices, we will obtain the undirected laplacian matrix (i.e., *L* = *L*^*h*^ + *L*^*l*^).

Once we have split the graph into two different ones, it is possible to get a generalization of the matrix representation of the consensus dynamics we had in Eq 3 that takes into account the potential asymmetry of the diffusion process,

$$\begin{array}{c} \begin{array}{ccc} \frac{dv}{dt} & = & -L^{l}\vec{v}\left(t\right)-\gamma L^{h}\vec{v}\left(t\right),\\ \Delta v(t) & = & -\eta L^{l}v\left(t-1\right)-\gamma \eta L^{h}v\left(t-1\right), \end{array} \end{array}$$ (5)

In Eq 5, parameter *γ* (0 ≤ *γ* < ∞) plays the role of the asymmetry factor. If *γ* = 1 we have no asymmetry and we are back into the undirected graph and standard diffusion process. However, if *γ* > 1 or *γ* < 1 we will have situations where the gradient is reduced at different speeds depending on their sign. In the following section, we will explore the consequences of these dynamics to the final equilibrium consensus in the network.

## Consensus in the generalized diffusion process

**Fig 1:**
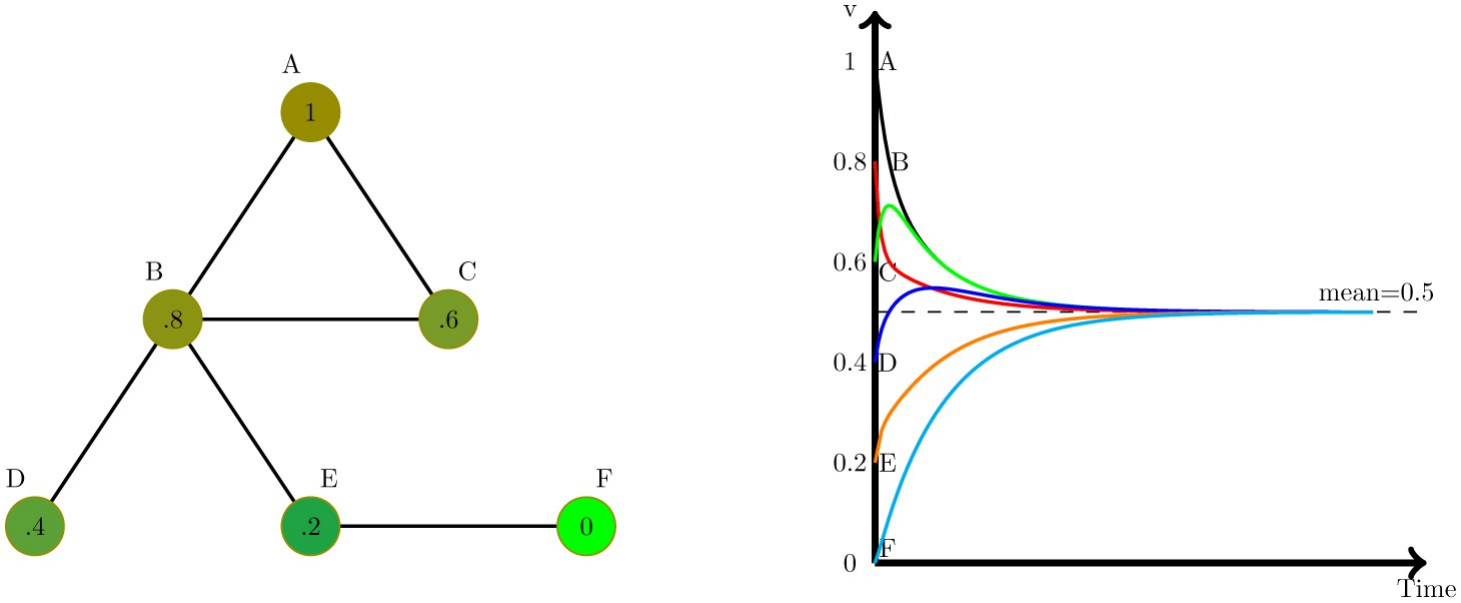
Simple undirected network and consensus, where each node has a different initial value of a variable of interest (left), and consensus dynamics (right).

To facilitate the description of the consequences of the generalization of the diffusion process we are going to use in this section a simple graph with six nodes (see Fig 1).

**Fig 2:**
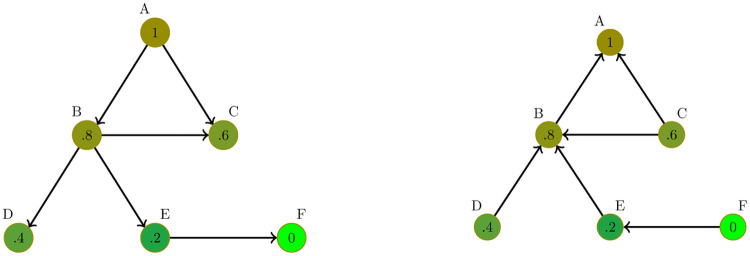
Directed networks representing the influence of high value nodes on low value nodes (left) and the influence of low value nodes on high value nodes (right).

The dynamical process defined by Eq 3 is linear, and has the expected consensus value in the average of the initial conditions (i.e., 〈*v*(0)〉) (see [35]), as shown in Fig 1. More interesting is what happens when we switch into the generalised diffusion process (Eq 5), where we have to divide the previous graph into two directional graphs, as shown in Fig 2.

**Fig 3:**
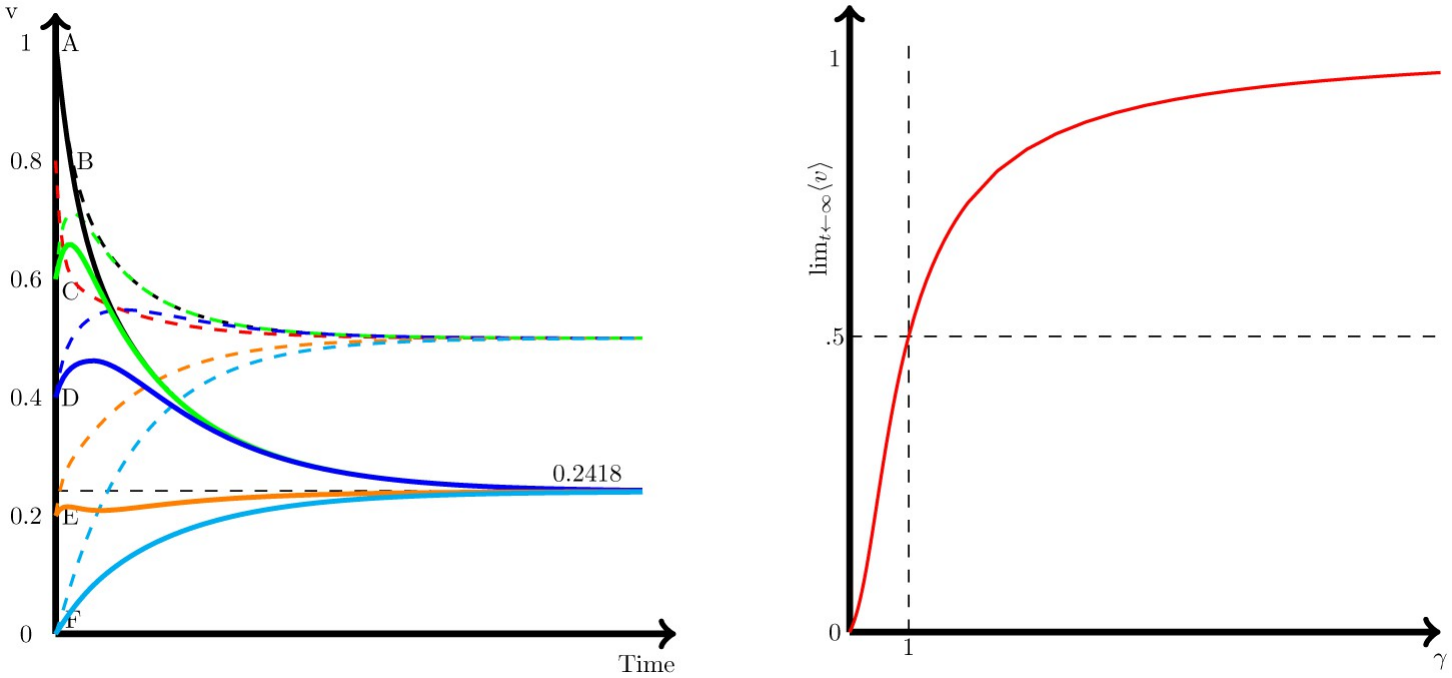
Solution for the asymmetric diffusion process 3. Left panel represents the solution for the network in Fig 2 and the dynamics of Eq 5 for *γ* = .5 in solid lines, and for *γ* = 1 in dashed lines, both with the same initial values (*u*_0_ = [1 .8 .6 .4 .2 0]^T^). Right panel represents the solution for different values of *γ* (0 ≤ *γ* < ∞).

The consensus generated in this case will be different from the simple case. One obvious reason is that since *γ* ≠ 1 produce different speeds of movement to consensus, the final agreement will be different from the average value of the initial conditions (lim_*t*→∞_*v*_*i*_(*t*) = *F*(*γ*) ≠ 〈*v*(0)〉). Fig 3 illustrates this. The left panel of Fig 3 represents how the dynamic evolution of *v* changes from the one in Fig 1 when *γ* = 0.5 instead of *γ* = 1. As we would expect since nodes with high values of *v* correct their gradient fastest than the nodes with low values, the final consensus is below the average of the initial values (lim_*t*→∞_*v*(*t*) = 0.2418 < 0.5 = 〈*v*(0)〉). Going to the extreme cases, if *γ* = 0, then the nodes with low values of *v* (i.e., negative gradient) do not move upward, while those nodes with high values of *v* (i.e., positive gradient) will reduce the values. As a consequence, the final consensus will be equal to the minimum of the initial values. Of course, if *γ* → ∞ the situation will be the opposite, and the final consensus will be closer to the maximum of the initial conditions (see Fig 3).

Other consequence of the dynamics of the search for consensus is that the gradient of the nodes changes sign with the pass of time (see Figs 1 and 3). This feature is irrelevant in the case of the symmetric diffusion process (i.e., when *γ* = 1). However, if *γ* ≠ 1, then any change in the sign of the gradient implies that there will be changes in *L*^*h*^ and *L*^*l*^ in different moments of time. That is, we will have both *L*^*h*^(*t*) and *L*^*l*^(*t*). Thus, a more adequate representation of the generalized diffusion process will be the one in Eq 6 rather than Eq 5, adding an additional layer of complexity to the dynamics of the system. In fact, this changing nature of the adjacency matrix makes no possible to obtain a closed form for the value of the final consensus.

$$\begin{array}{c} \begin{array}{ccc} \frac{dv}{dt} & = & -L^{l}\left(t\right)\vec{v}\left(t\right)-\gamma L^{h}\left(t\right)\vec{v}\left(t\right),\\ \Delta v(t) & = & -\eta L^{l}\left(t\right)v\left(t-1\right)-\gamma \eta L^{h}\left(t\right)v\left(t-1\right), \end{array} \end{array}$$ (6)

**Fig 4:**
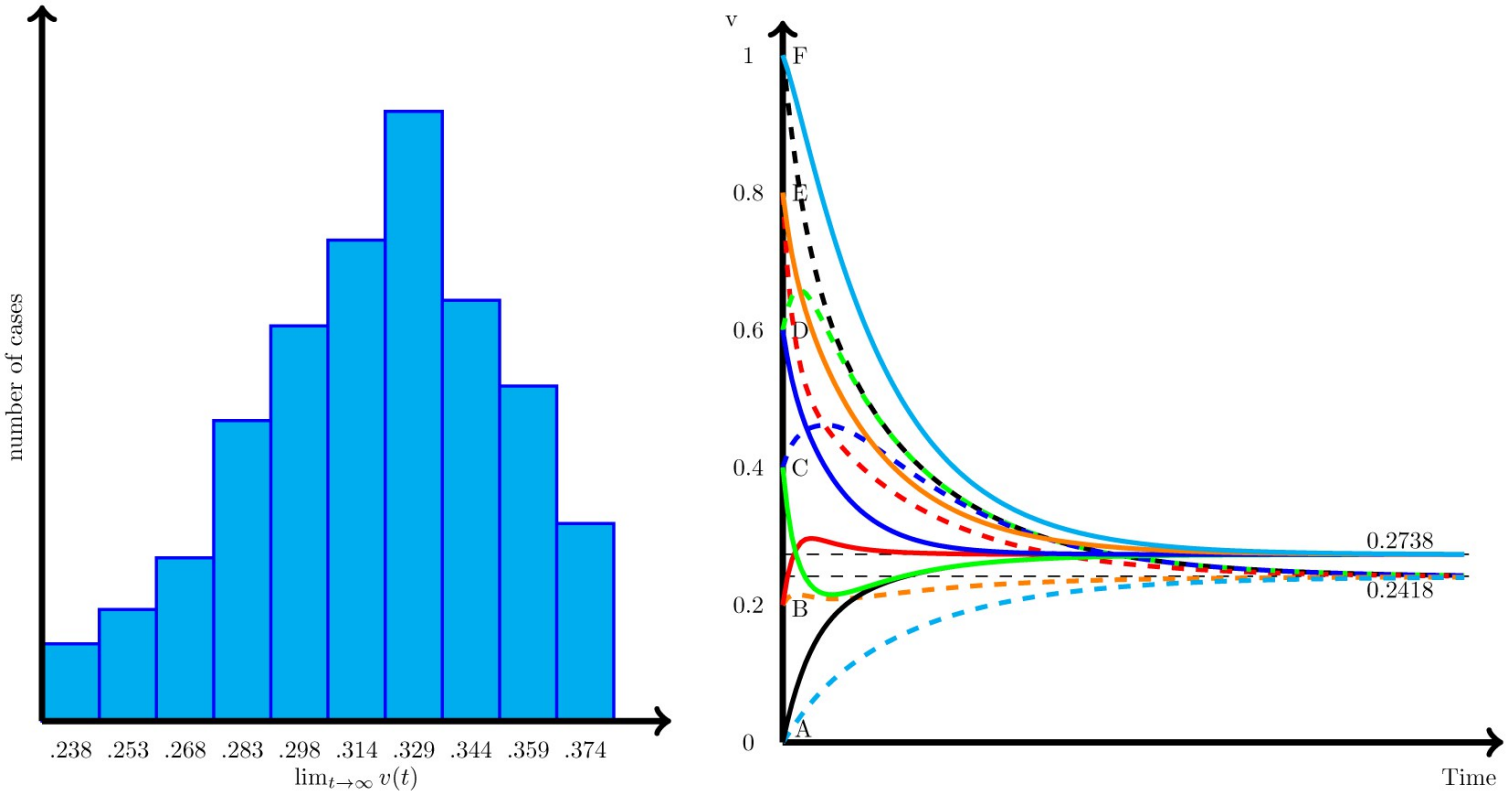
Effect of the distribution of initial values to the consensus. Solutions for the generalized diffusion process (Eq 6) for the graph represented by Fig 1, for *γ* = .5. Left panel represents, the consensus for all possible combinations of nodes and the initial values *u*_0_ = [1 .8 .6 .4 .2 0]^T^. Right panel shows the dynamic for two cases: *u*_0_ = [1 .8 .6 .4 .2 0]^T^ to *u*_0_ = [0 .2 .4 .6 .8 1]^T^.

Interestingly, the final consensus will also differ if we change the distribution of the initial values among nodes, even if they keep the same average 〈*v*(0)〉. Once more, in the symmetric case (i.e., *γ* = 1), this is not relevant. However, for the asymmetric case (e.g., *γ* = 0.5), if we move from *u*_0_ = [1 .8 .6 .4 .2 0]^T^ to *u*_0_ = [0 .2 .4 .6 .8 1]^T^, this change is enough to produce a final consensus that moves upward from 0.2418 to 0.2738 (see Fig 4, right panel). In fact, looking for the consensus for all possible combinations of the initial values *u*_0_ = [1 .8 .6 .4 .2 0]^T^ and nodes (i.e., 720 cases), we end with sizable differences in the consensus, which range from 0.23 to 0.38 (see Fig 4).

Given the considerable differences in the potential consensus, we explore which node is more relevant to the final consensus. In order to do so, we regress the value of this final consensus (*C*) with respect to the initial value of each node, as in Eq 7,

$$\begin{array}{c} C=\alpha +\beta_{B}*u_{B}\left(0\right)+\beta_{C}*u_{C}\left(0\right)+\beta_{D}*u_{D}\left(0\right)+\beta_{E}*u_{E}\left(0\right)+\beta_{F}*u_{F}\left(0\right)+\epsilon \end{array}$$ (7)

where *u*_*i*_(0) are the initial values of each node, *C* is the final consensus, *β* is the coefficient parameter for each node, *α* is the constant parameter, and *ϵ* is the error term.

The estimated value of each *β* in Eq 7 gives the relevance of each node in the final consensus *C*. The results of estimating the coefficients of this equation using the minimum squared error estimators are showed in Table 1. Since the sum of all initial values is always the same (to ensure the same average), there is perfect multicollinearity among the six nodes. Thus, we have to leave one of the nodes out of the regression to be able to estimate the coefficients. We have dropped node A from the regression because it is topologically equivalent to node C, and its inclusion would be redundant. Therefore, *β* coefficients must be interpreted as the differential effect of each node from the effect of node A on the final consensus. As can be seen in Table 1, nodes B, C, D and E have all of them the same influence as node A on the final consensus (i.e., none of the coefficients is statistically significant). However, the value of the initial conditions of node F has a highly statistically significant and positive effect on the final outcome. This might look counter-intuitive, since node F is precisely the node with the lowest centrality in the network (either in terms of Degree [36], Closeness [37] or Betweenness [36]), as Table 1 shows.

**Table 1:** Centrality measures and regression coefficients on the consensus of the graph diffusion for the 720 combinations of the initial values of the nodes. Figures in parentheses are the standard deviations of the estimated coefficients. *,**,*** indicates that the coefficient is statistically different from zero at the 10%, 5% and 1% significance levels.

Node	Coefficient (std.dev.)	Degree	Closeness	Betweenness
*α*	0.3068***			
	(.0089)			
*v*_*A*_(0)		2	0.1111	0
*v*_*B*_(0)	-0.0015	4	0.1667	8
	(.0045)			
*v*_*C*_(0)	-0.0000	2	0.1111	0
	(.0045)			
*v*_*D*_(0)	0.0010	1	0.1000	0
	(.0045)			
*v*_*E*_(0)	0.0018	2	0.1250	4
	(.0045)			
*v*_*F*_(0)	0.0217***	1	0.0833	0
	(.0045)			

**Fig 5:**
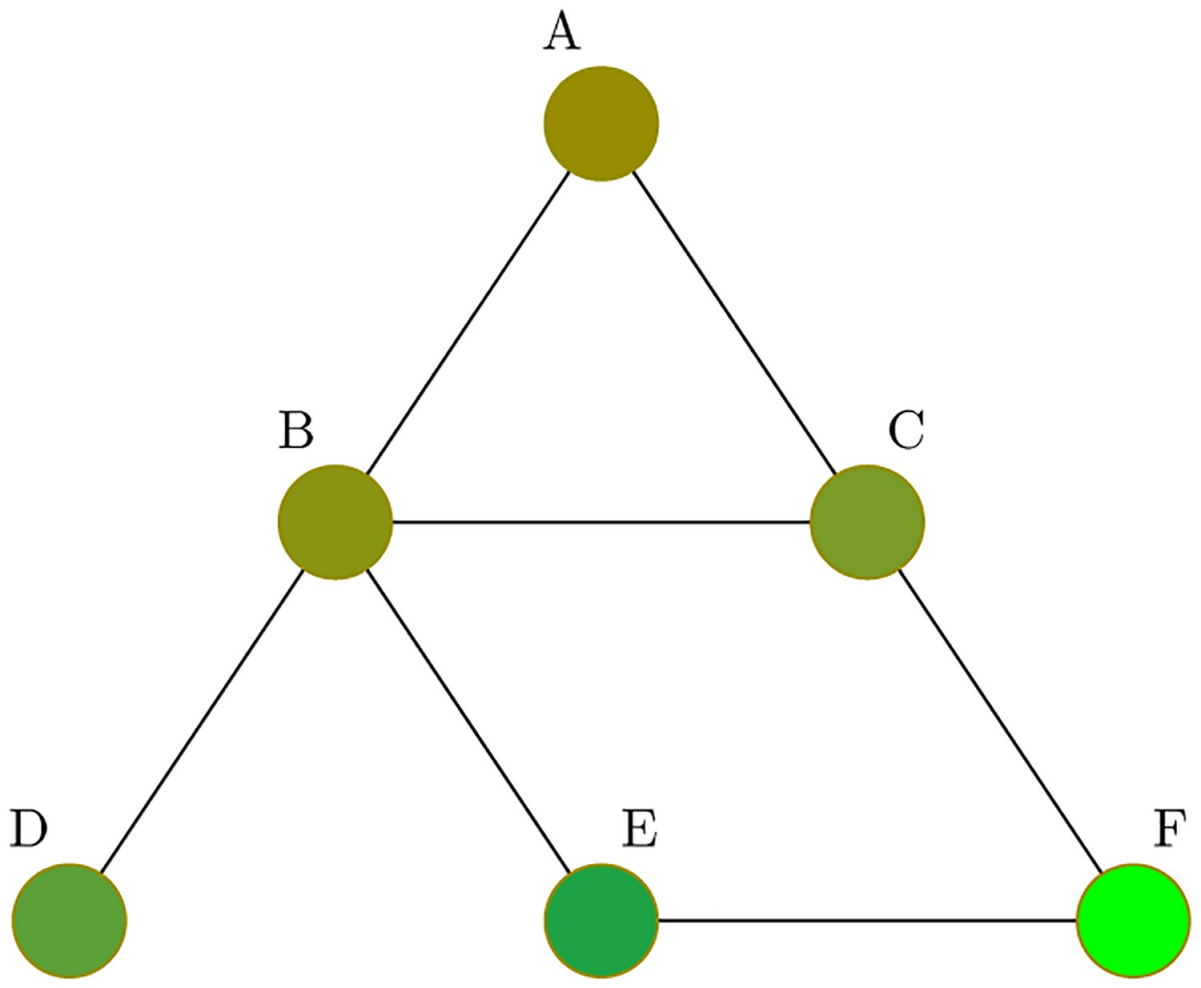
Alternative graph. By adding a link between node C and F to the graph of Fig 1, we are moving node F from the periphery of the graph to a more central position, leaving node D as the more eccentric node of the Graph.

Previous result points out to the relevance of the topology of the graph in the final outcome, and how the more eccentric nodes exert a high effect on the final consensus. The reason for this is that nodes that are at the center of the graph quickly converge to a consensus among them, while those in the periphery of the network are less influenced from other nodes in the graph. Therefore, in later stages, these peripheral nodes that are out of the general consensus continue moving the other nodes out of their previous consensus and closer to the periphery values. One way to test this interpretation is to look at the dynamics if we slightly change the topology of the graph to move node F from the periphery to a more central position in the network. In order to do so, we just need to add a new link between node F and node C (see Fig 5).

**Fig 6:**
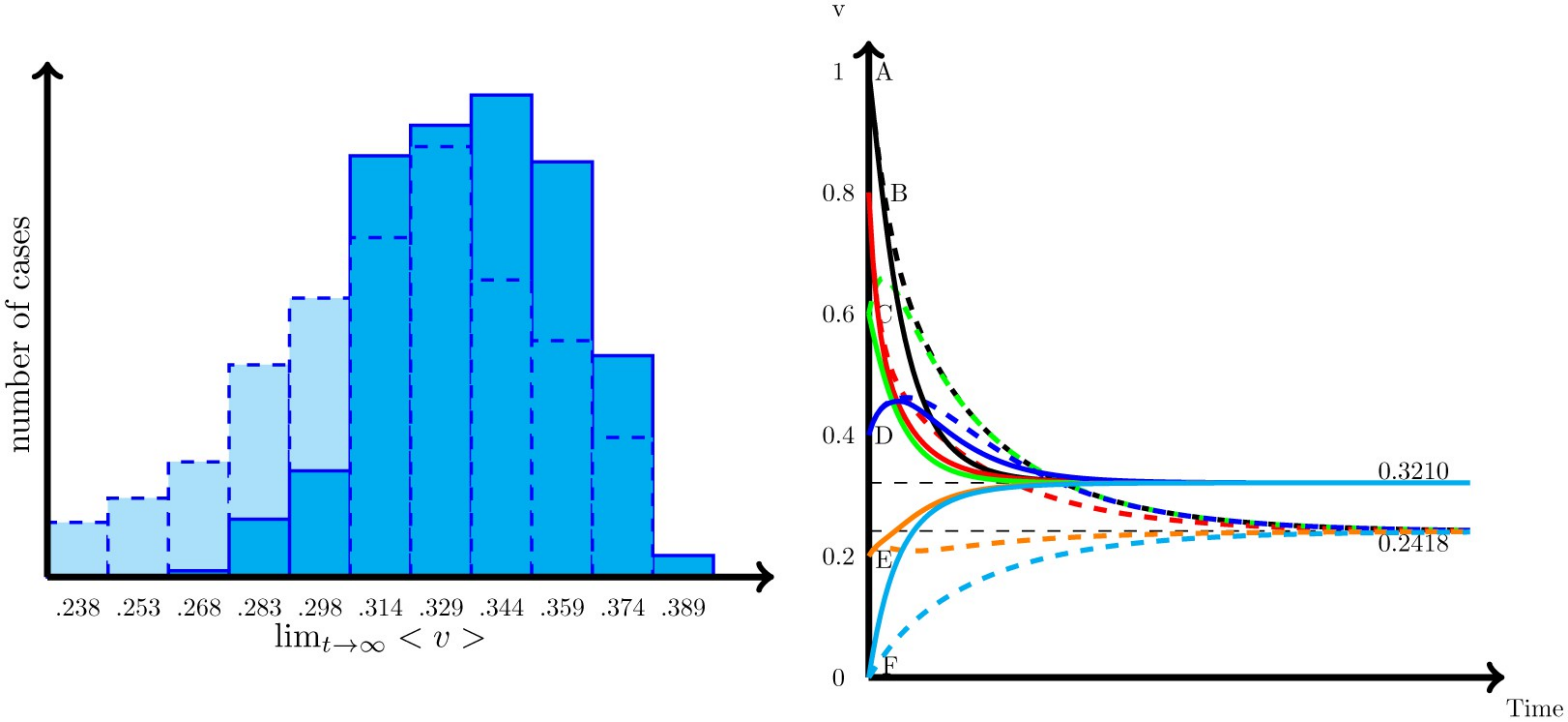
Sensitivity of the solution to the topology of the graph. Solutions for the generalized diffusion process with *γ* = 0.5 for the graph represented by Fig 5 in solid lines, and Fig 1 in dashed lines. Left panel shows the final consensus for both graphs and all 720 possible combinations of the initial values. Right panel shows the dynamics of both graphs for the case with initial values *u*_0_ = [.1 .05 .15 .20 .25 .1]^T^.

This minimum change in the topology of the network produce sizable effects on the final consensus of the graph as Fig 6 shows. In the original example of Fig 1, node F was the more eccentric node and also the one with the lowest initial value of *v*. When we compare the dynamics of that consensus with the one on the Fig 5 we observe that now that node F has a higher centrality in the graph the consensus move upwards (i.e., node F is not able to move the general consensus downwards as much as it did when F was in the periphery of the network). This is due to the different speed of convergence in both cases. While in Fig 1, F moved slowly to the consensus influenced only by a single node E, in Fig 5, there are two nodes (i.e., E and C) moving upward *v*_*F*_ and F quickly moves to converge to the other nodes. By contrast, node D is the one that now move at the lowest speed to the final consensus, since it is the one node remaining in the periphery.

To further confirm the intuition that the influence that node F had in the graph represented in Fig 1, has now moved to node D in the graph of Fig 5, we repeat the regression analysis of Eq 7. Thus, we compute the final consensus for the 720 combinations of initial conditions and nodes, and regress those consensus on the values of *v*(0) for each node. Results of the estimation of *β* coefficients via minimum squared error estimations are presented in Table 2. The regression analysis indeed confirms that node F no longer plays a significant role in the final consensus, now that has moved from the periphery to the center of the graph. That role is now played by node D, that is the node with the lowest centrality in the graph (Degree 1, Closeness 0.1 and Betweenness 0).

**Table 2:** Centrality measures and Regression coefficients on the consensus of the graph diffusion for the 720 combinations of the initial values of the nodes. Figures in parentheses are the standard deviations of the estimated coefficients. *,**,*** indicates that the coefficient is statistically different from zero at the 10%, 5% and 1% significance levels.

Node	Coefficient (std.dev.)	Degree	Closeness	Betweenness
constant	0.3325***			
	(.0064)			
*v*_*A*_(0)		2	0.1250	0
*v*_*B*_(0)	-0.0020	4	0.1667	5.5
	(.0032)			
*v*_*C*_(0)	-0.0025	3	0.1429	2
	(.0032)			
*v*_*D*_(0)	0.0143***	1	0.1000	0
	(.0032)			
*v*_*E*_(0)	-0.0018	2	0.1250	1
	(.0032)			
*v*_*F*_(0)	0.0006	2	0.1111	.5
	(.0032)			

To generalize the result obtained on the influence on the diffusion of the peripheral nodes we have performed the same analysis with four types of simulated larger networks: First, we have simulated a Erdős–Rényi (random) network with 200 nodes and a probability that there is a link between nodes of 5% [38]. The second network we have simulated is a Watts-Strogatz (small-world) network [39] of the same size (i.e., 200 nodes), so that each node is linked with the next 1–10 nodes (considering a random uniform distribution), and then, with a probability of 10%, the link created is deleted and changed by another link with another node that is any other place in the network. This way, we ensure that the network has both a high level of clusters, and also short paths between distant parts of the network. The third network simulated is one with a heavy tailed degree distribution (also size of 200 nodes), a reduced version of a scale free network [40] so that the probability that a new node is linked to the previous ones is a function of the degree of those nodes, such as the odd ratio of both nodes being linked increase a 10% with the degree (i.e., the nodes in the network have a degree *k* distribution: *p*(*k*)∼*k*^−2.9^). Finally, the fourth network is a core-periphery network [41], where half the nodes are in the core and have a 30% of being linked, while the other half (the periphery ones) have just a 0.1% probability of being linked, while the core and periphery have a 5% probability of being linked.

**Fig 7:**
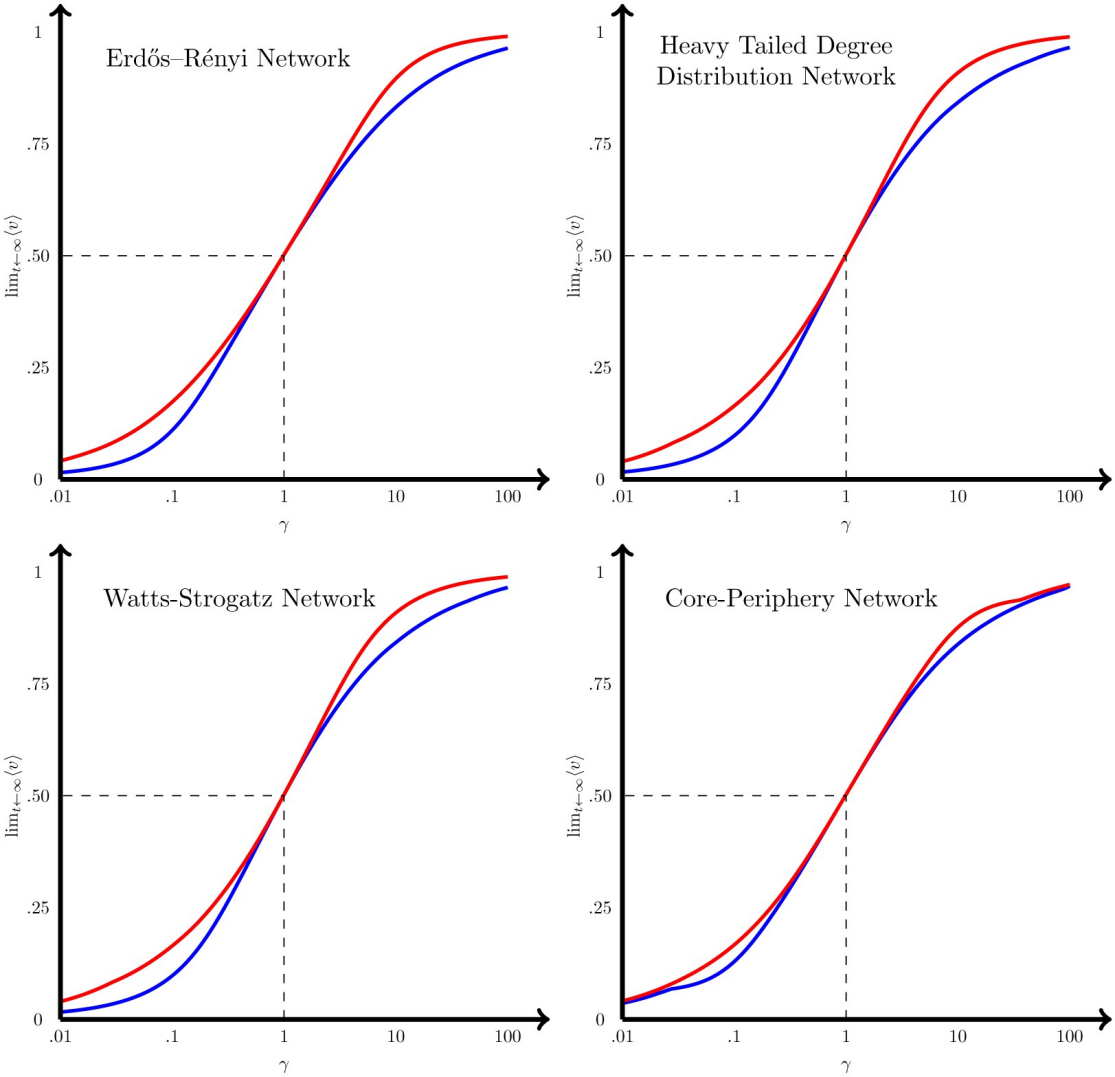
Solution for different type of networks, *γ* values and initial values. Solutions for a Erdös-Rényi network (top-left), Heavy-tailed degree distribution network (top-right), Watts-Strogatz network (bottom-left), and core-periphery network (bottom-right) and different values of *γ*, and initial values of *v* that are sorted to be directly correlated with the closeness of each node (blue) or the inverse of the closeness (red).

Once each network is built, the closeness (*C*) of all the nodes for each network are computed and the values ordered from lowest to highest, so equally separated values between 0 and 1 for the variable *v*(0) are assigned to each node *i* in ascending (*v*_*i*_(0) > *v*_*j*_(0) ⇔ *C*_*i*_ > *C*_*j*_), and descending order (*v*_*i*_(0) > *v*_*j*_(0) ⇔ *C*_*i*_ < *C*_*j*_). From there, we have studied the dynamics of the diffusion (see Fig 7). As can be seen, the ascending order configuration (where low closeness values imply low initial values), produce lower final solutions (blue lines) than where there is a descending order (red lines) configuration (where low closeness values imply high initial values), for any *γ* ≠ 1. This result further confirms that the initial values of the nodes in the periphery of a network (that we have defined as the nodes with lower closeness), are more relevant for driving the final solution up or down, than the initial values of the nodes in the core of the network.

## An empirical application: The proportion of women on company boards

We show in this section an empirical application of the generalized diffusion process we have introduced in the previous section in the network of boards and directors from US listed companies, where the variable diffused through the network is the proportion of women appointed as directors.

### Company boards as a complex network

The board of directors is at the top of the hierarchy of a company. Shareholders entrust the board of directors to defend their interests. Boards are composed by executive and independent directors. Executive directors act also as part of the top management team and have inside knowledge and responsibility in the company. Independent directors are members of the board that supervise shareholders’ interests and represent them at the board discussion. These directors usually show a part-time commitment with the company. Therefore, while executive directors have an exclusive relationship with boards, independent directors can serve to multiple boards simultaneously.

These links of independent directors with several corporations constitute a network in which each board is a node, and nodes are connected when they share directors. These networks have been explored in the past (e.g., [42–44]), although, to our knowledge, this is the first time to be analyzed within the context of a dynamic continuous diffusion process.

Using data from BoardEx database, we use a panel composed by 9,891 US listed companies, and their directors from 2000 to 2015. Using each year’s board composition, we can build a network for each year, composed by the directors serving on more than one board (e.g., in 2015, we found 29,060 board connections).

**Fig 8:**
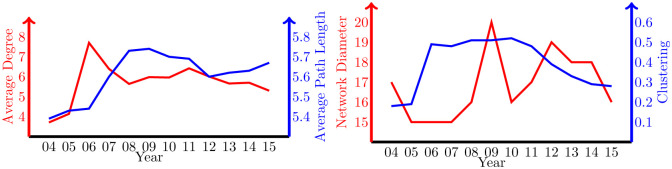
Network properties. The average degree (red, left scale) and average path length (blue, right scale) in the left panel. The network diameter (red, left scale) and clustering (blue, right scale) in the right panel.

This network presents some characteristics of a small world. Fig 8 summarizes some of its key characteristics. The average degree indicates that each board is connected to, on average, 5 to 8 other boards. The total diameter ranges from 13 to 20, this being the number of steps between the two most distant boards. However, the average path length is around 5, meaning that, on average, only 4 steps (directors) are needed to join two boards. The clustering reflects the likelihood of two nodes being connected if they share a mutual neighbor, and is determined by the relative number of triangles in the graph: if one board is connected with two other boards, the clustering measures whether they are also connected to each other. Small average path length and high transitivity are two properties that are usually present in small-world networks.

**Fig 9:**
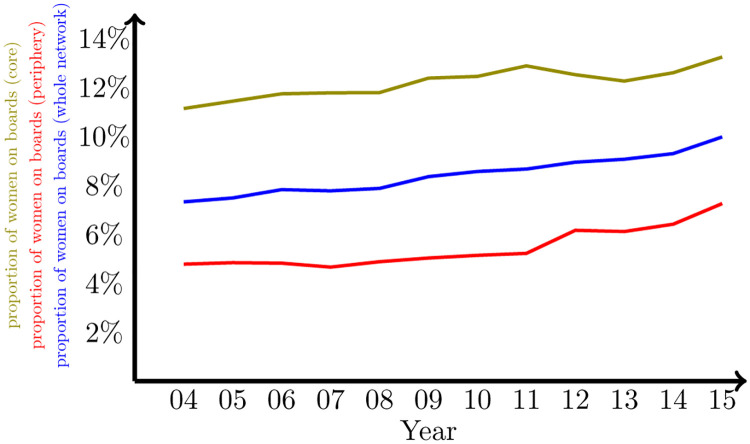
Proportion of women on boards. Average proportion of women on the whole network (blue), on the third boards with the lowest closeness centrality scores (red), and on the third boards with the highest closeness centrality scores (olive).

The variable of interest in our analysis is the percentage of women appointed to work on each board (*ω*). The scarcity of women on boards has received considerable attention by researchers research (e.g., [45–47]), policy-makers, investors, and other stakeholders ([48, 49]). *ω* is considered by several actors as an important source of corporate legitimacy. This creates pressure among corporations to emulate its neighboring boards. The evolution of *ω* have been upward from a 7.3% in 2004 to a 9.96% in 2015 (see Fig 9), well below the values that you would expect from a group that represents half the population.

A feature of special interest in our analysis of a generalized diffusion process is the way the variable of interest (i.e., *ω*) is distributed throughout the network. As Fig 9 shows, a persistence feature is that the proportion of women is higher in the core of the network, measured by the closeness of each board (i.e., goes from 11.1% in 2004 to 13.2% in 2015, for those boards in the first third of the highest closeness scores), than in the periphery (from 4.8% to 7.2% in the same period for those boards in the third of the network with lowest closeness scores). As we explained in the previous section, the consensus value in a context of asymmetric diffusion is overweightedly determined by the values of those nodes on the periphery. In this case, the periphery is dominated by an even lower proportion of women on boards. Therefore, regardless of the sign of the asymmetry, if this is present, this will imply that the periphery will pressure the whole network for a lower proportion of women.

### Empirical diffusion model

We propose the following model for the diffusion of the proportion of women on boards (i.e., *ω*_*i*_ for company *i*),

$$\begin{array}{c} \begin{array}{cc} \Delta \omega_{i}\left(t+1\right)=\alpha & -\beta_{l}\sum_{j}a_{ij}^{l}\left(t\right)\frac{1}{k_{i}\left(t\right)}(\omega_{i}\left(t\right)-\omega_{j}\left(t\right))\\ & -\beta_{h}\sum_{j}a_{ij}^{h}\left(t\right)\frac{1}{k_{i}\left(t\right)}(\omega_{i}\left(t\right)-\omega_{j}\left(t\right))+\epsilon_{it}, \end{array} \end{array}$$ (8)

The left hand side (Δ*ω*_*i*_(*t* + 1)) represents the change in the proportion of women (*ω*) in company *i* between consecutive years *t* and *t* + 1. On the right side, $a_{ij}^{l}\left(t\right)$ and $a_{ij}^{h}\left(t\right)$ are the coefficients of the directed networks, so $a_{ij}^{l}\left(t\right)=1$ if boards *i* and *j* share a director and *ω*_*j*_(*t*) < *ω*_*i*_(*t*), and $a_{ij}^{h}\left(t\right)=1$ if boards *i* and *j* share a director and *ω*_*j*_(*t*) > *ω*_*i*_(*t*), and zero otherwise; *k*_*i*_ is the degree of board *i*; and *ν*_*it*_ is the random noise including all other determinants of *ω*_*i*_(*t* + 1) that are non-related to the diffusion process.

The difference in the proportion of women between boards *i* and *j* (i.e., gradient *ω*_*i*_(*t*) − *ω*_*j*_(*t*)). The diffusion is determined by parameters *β*_*l*_ and *β*_*h*_. if either *β*_*l*_ > 0 or *β*_*h*_ > 0, we can affirm that there is diffusion in the network, and companies copy each other in the value of *ω*. By contrast, if both *β*_*l*_ = *β*_*h*_ = 0, it would imply that there is no diffusion. In the case of *β*_*l*_ = *β*_*h*_ > 0, then we are in a situation of a symmetric diffusion. While if *β*_*l*_ > *β*_*h*_, there is a situation where the reduction in the gradient is faster for those companies that have a higher proportion of women on their boards than their neighbors than in the opposite situation. Conversely, if *β*_*h*_ > *β*_*l*_, the speed of increase is higher than the speed of reduction.

In any case, we cannot assume that the network is a closed system, since new directors are appointed (i.e., new hiring) and leave (i.e., either for retirement, lay off, or death) the network constantly. To reflect this exchange between the network and an external director’ pool, we include *α* to the model in line with what you would have in a heat diffusion.

**Fig 10:**
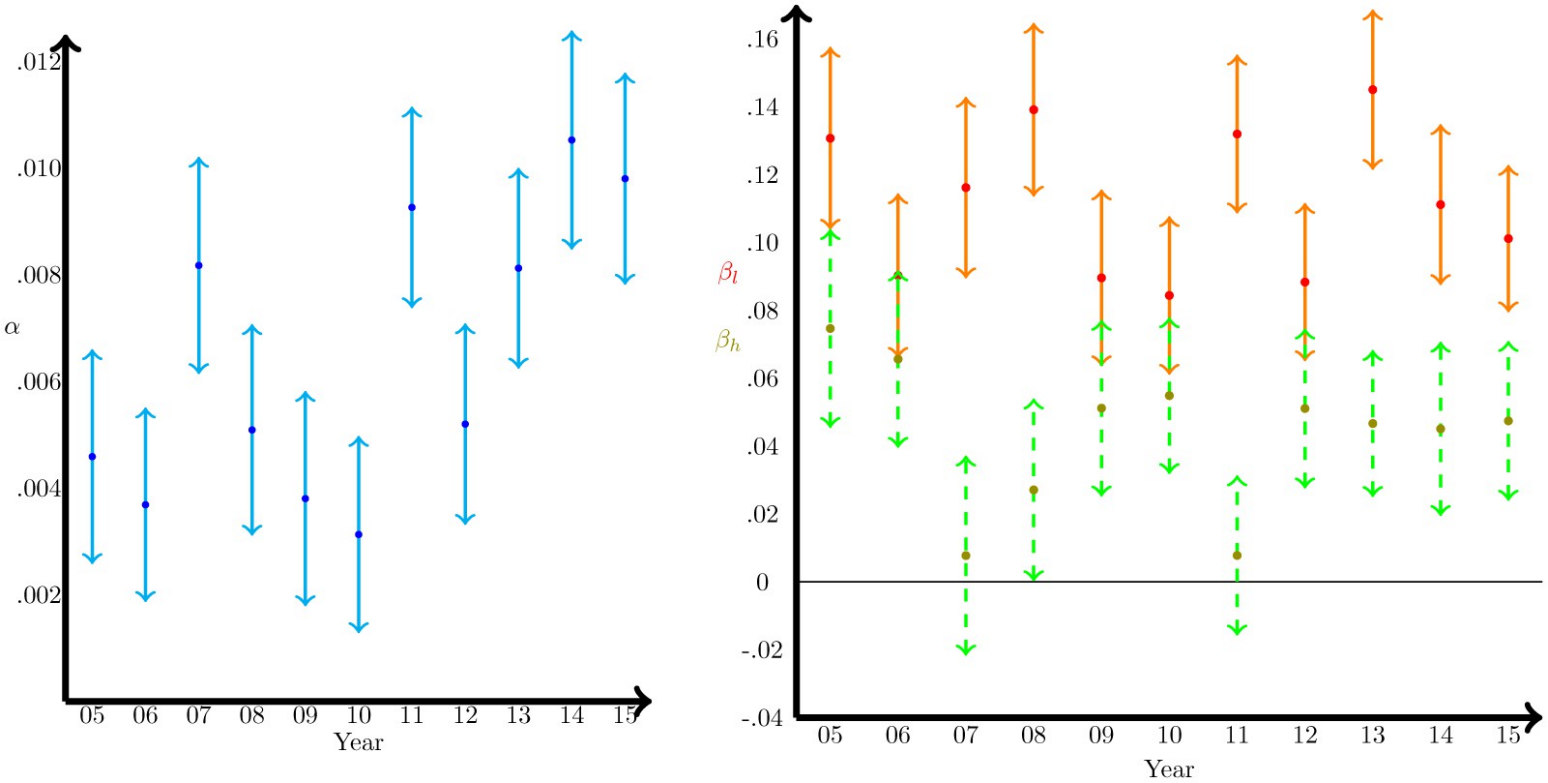
Parameter estimates for the empirical model. Left panel represents the estimated parameter *α* of annual increase of *ω* across US Boards. Right panel represents the estimated *β*_*h*_ and *β*_*l*_ parameters for the diffusion of *ω*. All parameters are estimated using minimum squared error estimators, with 95% confidence intervals.

Since we observe *ω*_*i*_(*t*), we can recover by means of minimum squared errors regression analysis the estimated values of *α*, *β*_*h*_ and *β*_*l*_ in Eq 8 for each year. Estimated values for each year are shown in Fig 10 along with their 95% confidence intervals. The estimated parameters are relatively homogeneous between the years considered and always clearly above zero. This implies that there is a diffusion of *ω* and that there is clearly an asymmetry in the diffusion. The values obtained for *β*_*l*_ are systematically higher (approximately double) than for *β*_*h*_. Therefore, diffusion runs faster when the company has a higher value of *ω* than its neighbors and there are pressured to reduce *ω*, and lower when the company has a lower value of *ω* than its neighbors and are pressured to increase the value of *ω*.

**Fig 11:**
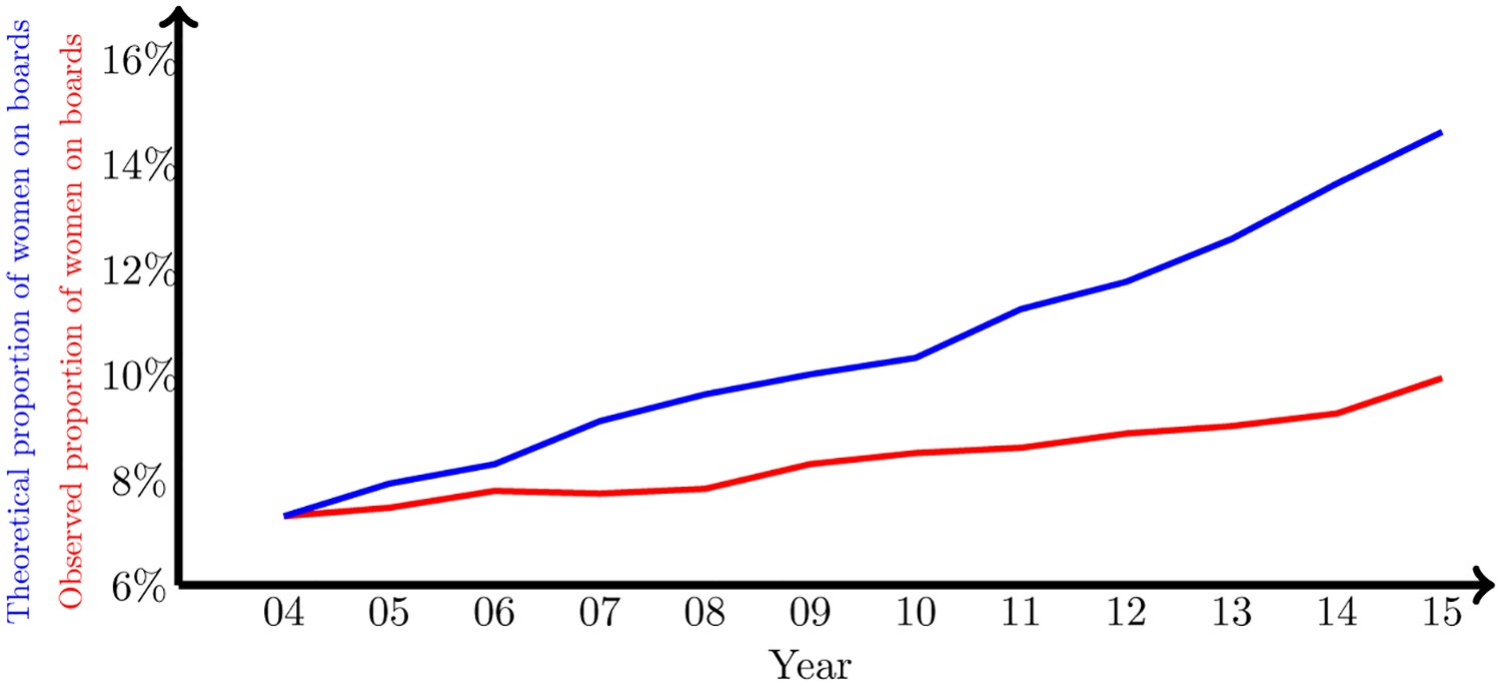
Counterfactual evolution of the average proportion of women on bards. Evolution of the observed average proportion of women on boards (red), and counterfactual evolution of the same variable in the absence of asymmetry in the diffusion (blue).

The constant *α* is also positive and significant, implying an annual increase in the proportion of women on boards between 0.3% and 1% (see Fig 10) as a consequence of the replacement of older generations of directors with younger ones, where the representation of women is higher. However, although the open nature of the network produces an overall increase in the proportion of women directors (*α* > 0), the asymmetry of the diffusion parameters (i.e., *β*_*l*_ > *β*_*h*_) creates a pressure in the graph to keep the proportion of women on boards at low levels—hence maintaining the same presence of men, what has been known as the Old Boys’ Club ([45, 47]). In fact, by accumulating the estimated *α* parameters for the 12 years studied, we can evaluate the effect of the downward pressure of the asymmetric diffusion dynamic in the network, since in case of *β*_*l*_ = *β*_*h*_, the only relevant magnitude for the evolution of the mean *ω* would be *α*. The result of this exercise is precisely what we show in Fig 11. According to this, the average proportion of women, that went from 7.43% to 9.93%, would have go to 14.61% without the asymmetry.

## Conclusions

In this paper, we show how asymmetries in the consensus dynamics produce the emergence of non-linear dynamics in the diffusion processes of graphs and networks that are otherwise linear.

We use a double layer of directional graphs to show how this behavior can arise. In this case, a complex asymptotic solution of the problem appears depending on a) differences in the diffusion rate of opposing forces, b) the initial conditions, and c) the graph topology. The analysis here developed could be considered a generalization of other particular cases such as the symmetric case, or the zealot problem. One interesting result is that the nodes in the periphery of the network are more relevant to the final consensus than the nodes in the core of the network.

The illustration on this asymmetry can be seen in the behavior of the diffusion of gender diversity on boards in the US board network. This example shows how this asymmetry works: when a company has a higher presence of women on board than neighboring companies, the speed of reduction is faster than in the opposite situation. As a consequence, this asymmetry leads to a lower asymptotic consensus value, turning into a long-standing scarcity of female representation on boards, that we evaluate in 5 percentage points between 2004 and 2015 (i.e., from 14.6% to 9.9%).

This research is very relevant, as in the diffusion of any other characteristic through a network, can also arise asymmetries if the resistance to change in one direction is higher than in the other, deserving a closer analysis. These can include networks from epidemiology to Twitter discussions. Therefore, it is of high interest to consider asymmetries into the analysis of networks in order avoiding forecasting errors and wrong results.

Furthermore, future analysis could delve deeper into the case of considering weights in the model, considering for example the cases when boards share members. Other future work would be analyze whether it could be possible to change the speed needed to arrive to a consensus by means of shocks of different kinds on the network (i.e. regulations in terms of rate of woman in the boards).

## Supporting information

S1 File (ZIP)

S2 File (ZIP)
